# Oral administration of *Lactobacillus* reuteri GMNL-263 improves insulin resistance and ameliorates hepatic steatosis in high fructose-fed rats

**DOI:** 10.1186/1743-7075-10-35

**Published:** 2013-04-17

**Authors:** Feng-Ching Hsieh, Chia-Lin Lee, Chee-Yin Chai, Wan-Tzu Chen, Ying-Chen Lu, Ching-Shuang Wu

**Affiliations:** 1 Department of Medical Laboratory Science and biotechnology, Kaohsiung Medical University, 6F, Chi-Shih Building, 100, Shih-Chuan 1st Road, Kaohsiung, 80708, Taiwan; 2 Department of Research and Development, GenMont Biotech. Inc, Tainan, Taiwan; 3 Department of Pathology, Kaohsiung Medical University Hospital, Kaohsiung, Taiwan; 4 Department of Biological Science and Technology, Chung Hwa University of Medical Technology, Tainan, Taiwan

**Keywords:** *Lactobacillus reuteri* GMNL-263, High-fructose-diet, Type 2 diabetes, Insulin resistance, Hepatic steatosis

## Abstract

**Background:**

Type 2 diabetes mellitus (DM), characterized by peripheral insulin resistance, is the most common form of diabetes. Probiotics are live micro-organisms that, when administered in adequate amounts, confer delaying effect on DM development. In this study, the effects *Lactobacillus reuteri* GMNL-263 (Lr263), a new probiotic strain developed by our laboratory, on insulin resistance and the development of hepatic steatosis in high-fructose fed rats were explored. Furthermore, the relevant regulatory pathways involved were also investigated.

**Method:**

Male Sprague–Dawley rats were fed a high-fructose diet with or without Lr263 administration for 14 weeks. The composition of fecal microbiota, oral glucose tolerance, glycated haemoglobin, insulin, leptin, C-peptide, and incretins were measured. The markers of liver injury, serum and hepatic lipids profile, activity of hepatic antioxidant enzyme, and proinflammatory cytokines in adipose tissue were investigated. Additionally, the expression of hepatic lipogenic genes and insulin signaling related genes in adipose tissue were also studied. Liver sections were examined for hepatic steatosis using hematoxylin-eosin staining.

**Results:**

The levels of serum glucose, insulin, leptin, C-peptide, glycated hemoglobin, GLP-1, liver injury markers, lipid profile in serum and liver were significantly increased in high-fructose-fed rats. However, after Lr263 administration, the elevation of these parameters was significantly suppressed. Feeding of Lr263 reversed the decreased number of *bifidobacterium* species and *lactobacillus* species and increased number of *clostridium* species induced by high fructose treatment. The decreased activities of hepatic antioxidant enzymes in HFD rats were dramatically reversed by Lr263 treatment. Concentrations of IL-6 and TNF-α in adipose tissue which were elevated in high fructose treatment were markedly decreased after Lr263 feeding. Decreased levels of PPAR-γ and GLUT4 mRNA after high fructose treatment were significantly enhanced by Lr263 administration. Lr263 consumption normalized the increased lipogenic gene (Srebp-1c, FAS, and Elvol6) expressions stimulated by high fructose. Administration of Lr263 significantly ameliorated hepatic steatosis observed in high fructose treated rats.

**Conclusion:**

Our study provided evidences clarifying the effectiveness of Lr263 on reducing insulin resistance as well as hepatic steatosis formation in high-fructose-fed rats and suggested that Lr263 may be a promising therapeutic agent in treating type 2 diabetes.

## Background

Diabetes is a chronic global health problem, however, its etiology still remains elusive. The development of diabetic complications is a major cause of premature morbidity and mortality in diabetic patients [1]. There are two main forms of diabetes namely type 1 diabetes and type 2 diabetes [2]. Type 1 diabetes is typically caused by an autoimmune assault against the β-cells, resulting in lack of insulin secretion [3]. Type 2 diabetes is the most common form of diabetes comprising about 80% of all diabetic population characterizing by insulin resistance, dyslipidemia, and hyperglycemia [4-6]. The World Health Organization has predicted that developing countries would have to bear the major burden of this disease. It has been estimated that there will be a 42% increase from 51 million individuals in 1995 to 72 million individuals in 2025 affected in the developed countries [7]. However, in developing countries these figures are much higher and are expected to show 170% increase from 84 to 228 million [7]. Insulin resistance, a major abnormality underlying type 2 diabetes mellitus, is defined as the pathophysiological condition reducing insulin responsiveness in liver, muscle, and adipose tissue [8,9]. Aberrations of metabolism caused by insulin resistance may increase levels of circulating free fatty acids and liver fat accumulation, which then leads to liver inflammation and fibrosis [10]. Furthermore, high levels of free fatty acids induce the overexpression of cytochrome P450 (CYP) 2E1 and increase oxidative stress via lipid peroxidation, which may lead to liver injury [11].

Intestinal flora has been shown to influence human health such as immuno-stimulation, improved digestion and absorption, vitamin synthesis, inhibition of the growth of potential pathogens, cholesterol reduction, and lowering of gas distension [12-14]. The best way for controlling the flora balance in intestine is achieved by intake of probiotics, which is thought to be effective in lifestyle-related diseases [12,13]. Probiotics are defined as nonpathogenic live microorganisms that, when ingested adequately, confer health benefits to the host. The exact mechanisms by which probiotics exert their effectiveness on diabetes and its related complications are still inconclusive. Recently, probiotics have been reported to prevent or delay the onset of diabetes in various experimental models including chemical-induced, diet-induced, and genetically mutated animals [15-18]. Among these probiotics, *Lactobacillus* species, constituting the major part of the lactic acid bacteria group, have been reported to exhibit prominent effects on the management of diabetes and its complications. More specifically, oral administration of *Lactobacillus casei* exhibited a preventive effect on the elevation of plasma glucose and reduction of plasma insulin level resulted from destruction of pancreatic *β*-cells in Non-obese diabetic mice [15]. Yadav et al. [19] demonstrated that, Dahi (containing probiotic *Lactobacillus acidophilus* and *Lactobacillus casei*), a traditional India fermented milk product, demonstrated an inhibitory effect on development of glucose intolerance, hyperglycemia, hyperinsulinemia, dyslipidemia, and oxidative stress in high fructose-induced diabetic rats. The same treatments significantly suppressed the depletion of insulin as well as preserving diabetic dyslipidemia, and inhibited lipid peroxidation and nitrite formation in streptozotocin-induced diabetic rats [20]. Furthermore, *Lactobacillus GG* displayed an antidiabetic effect on neonatal streptozotocin-induced diabetic rats by lowering the blood HbA_1c_ level and improving glucose tolerance [17]. These results implied that administration of different *Lactobacillus* strains may confer antidiabetic effect as well as delaying diabetes onset.

*Lactobacillus reuteri* GMNL-263 (Lr263) is a representative probiotic bacteria that is commercially available as a healthy food in Taiwan. Our previous study demonstrated that oral administration of Lr263 has a preventive effect on the elevation of plasma glucose and renal fibrosis in streptozotocin-induced diabetic rats [21]. However, the effects of Lr263 on type 2 diabetes and its related complications remained unraveled. Thus, the present study was set out to explore the effect of oral administration of Lr263 on insulin resistance and the associated metabolic syndrome using a well-established animal model of type 2 diabetes characterized by insulin resistance, metabolic syndrome, and oxidative stress. Furthermore, the possible regulatory pathways involved were also investigated.

## Materials and Methods

### Preparation of *Lactobacillus reuteri* GMNL-263

Lr263 was obtained from the culture collection of the GenMont Biotech Incorporation (Taiwan). *Lactobacilli* microorganisms for oral administration were prepared from overnight cultures of *Lactobacilli* and subcultured in Man Rogosa Sharpe (MRS) broth (Difco Laboratories, Detroit, MI, USA) at 37°C. These microorganisms were collected by centrifugation to remove the MRS broth and suspended in sterile water at a concentration of 10^9^ CFU/ml.

### Animal model

Male Sprague–Dawley rats (BioLASCO Taiwan Co., Ltd., Yi-Lan, Taiwan) were housed in plastic cages in an animal room maintained with a 12 h light–dark cycle at 24 ± 1°C and 50% humidity. Animals were allowed free access to water and food throughout the study. The animals used in the present study were cared for according to the principles and guidelines of the Institutional Animal Ethical Committee (IAEC). All procedures were approved by the IAEC.

### Experimental design

Animals used in the study were divided into (1) control group (Control; n = 6) that consumed a standard 65% cornstarch diet (PMI Nutrition International, Brentwood, MO, USA), (2) high fructose-diet group (HFD; n = 6) that consumed a 65% fructose diet (Harlan Laboratories, Inc., California, USA), and (3) HFD + Lr263 group (n = 6) which were simultaneously fed with the 65% fructose diet and Lr263 (2 × 10^9^ CFU/rat) every day for 14 weeks except for the days before oral glucose tolerance test (OGTT) and blood collection. Lr263 was administered orally to rats by an oral gavage.

### Body weight and food consumption

The body weight of each rat was measured pre-test, weekly thereafter and at sacrifice after fasting. Food consumption for each rat was determined weekly.

### Enumeration of feces microbiota using plate culture

The cultivation of intestinal microbiota was performed according to the method previously described by Montesi et al. [22] with modifications. Briefly, fresh fecal samples were collected in sterile tubes and stored in aliquots at −80°Cfor a maximum of 1 month until processing. Samples were homogenized in a sterile peptone-salt solution (enzymatic digest of casein 1 g/L, sodium chloride 8.5 g/L). Serial 10-fold dilutions were prepared and volumes of 100 μl of 3 appropriate dilutions were loaded onto the surface of plates in duplicate which contained the following agar media: Wilkins-Chalgren agar (Oxoid, Basingstoke, UK) supplemented with MRS (Oxoid) for bifidobacteria and lactobacilli; supplemented with SPS (Oxoid) for clostridia. All these plates were incubated anaerobically at 37°C for 4–5 days. Representative colonies of each selective medium were identified to genus level by standard bacteriological methods including Gram stain, morphology, and biochemical reactions. The number of colony counting for each microbiota species was expressed as log CFU/g fecal content.

### Blood and tissue sample collection

At the end of the experiments, the animals were sacrificed by CO_2_ anesthesia. The blood samples were collected and centrifuged at 4000 × *g* for 10 min at 4°C and then stored at −80°C. Liver, adipose tissue, and kidney were weighted and washed with ice-cold saline, then were stored at −80°C until used.

### Oral glucose tolerance test

Oral glucose tolerance tests were performed at 0, 4, 8 and 14 weeks after treatments. The diets were removed from animal cages for 12 h before the administration of an oral glucose load (2 g/kg of body weight) by an oral gavage. Blood samples were collected from the tail vein at 0, 15, 30, 60, 90, and 120 min after glucose administration. Glucose concentrations were determined by glucometer (TaiDoc Technology Co, Taipei, Taiwan). The total glucose areas under the curve (AUC_glucose_) between 0 and 120 min represented the magnitude of the glucose response and were calculated as described previously [23].

### Measurement of serum/plasma glucose, insulin, leptin, C-peptide, active Glucagon-like peptide-1 (GLP-1), and total Gastric inhibitory polypeptide (GIP) concentrations

Serum glucose concentrations were determined by glucometer (TaiDoc Technology Co, Taipei, Taiwan). Serum levels of insulin, leptin, and C-peptide, plasma levels of active GLP-1 and total GIP were measured by commercial ELISA Kit (Millipore, MA, USA) according to the manufacturer’s instructions. For the measurement of active GLP-1 and total GIP, blood samples were collected and immediately subjected to plasma separation in centrifuge tubes containing EDTA-2Na and aprotinin. The detection limits of insulin, leptin, C-peptide, GLP-1, and GIP assays were 0.2 ng/ml, 0.2 ng/ml, 25.0 pM, 4.1 pM, and 8.2 pg/ml, respectively.

### Measurement of HbA1c

The whole blood samples of experimental rats at week 0, 4, 8, and 14 were collected and subjected to HbA1c determination. The levels of HbA1c were measured by the DCA-2000 system (Ames, Bayer Diagnostics, Basingstoke, England). The method was based on immunological detection of glycated Hb. The monoclonal antibody used was specific for an NH2-terminal amino-acid sequence in the beta chain of glycated Hb, and thus did not bind to other glycated Hbs such as HbAla or HbAlb. Both glucose and this specific amino-acid sequence were necessary for antibody binding. The monoclonal antibody was supplied bound to latex beads and was agglutinated by the second reagent in the absence of HbAlc. Glycated Hb thus competed with the agglutinating agent for antibody binding sites, thereby blocking agglutination. This agglutination reaction caused increased light-scattering which was measured as an increase in absorbance at 531 nm. The amount of HbAlc was calculated as a fraction of total Hb. Ratio of HbA1c to total hemoglobin was calculated and reported directly as % HbA1c.

### Biochemical parameters analysis

Serum biochemical parameters including triglyceride (TG) (detection limit was 3 mg/dl), low density lipoprotein (LDL) (detection limit was 5 mg/dl), cholesterol (detection limit was 3 mg/dl), aspartate aminotransferase (AST) (detection limit was 0.04 U/dl), and alanine aminotransferase (ALT) (detection limit was 0.04 U/dl) were analyzed using an automatic clinical analyzer (Hitachi High-Technologies Corporation, Tokyo, Japan).

### Measurement of hepatic antioxidants

Liver samples were collected and homogenized in 4 volumes of 56 mM Tris/HCl buffer (pH 7.4) containing 1.15% potassium chloride and then centrifuged at 10,000 g for 15 min. The supernatants were collected and stored at −80°C. The concentrations of protein in supernatants were determined using a commercial kit (Bio-Rad) according to the manufacturer’s instructions. The activity of superoxide dismutase (SOD) and glutathione reductase (GR) in the liver were evaluated using commercial SOD assay kit (Sigma), and glutathione reductase assay kit (Sigma), respectively, according to the manufacturer’s instruction. The enzyme activity of SOD and GR were expressed as U/mg protein. The detection limit of SOD and GR assay were 0.001U/ml and 0.003 U/ml.

### Measurement of TNF-α and IL-6 levels in adipose tissue

The adipose tissues (0.1-0.3 g) were homogenized in RIPA buffer (0.625% Nonidet P-40, 0.625% sodium deoxycholate, 6.25 mM sodium phosphate, and 1 mM ethylenediamine tetraacetic acid at pH 7.4) containing 10 μg/ml of a protease inhibitor cocktail (Sigma, St. Louis, Missouri, USA). Homogenates were centrifuged at 12,000 g for 10 min at 4°C. Then the supernatants were collected and subjected to protein concentration determinations using a commercial kit (Bio-Rad DC Protein Assay, Bio-Rad, CA, USA). Quantitative assessment of TNF-α and IL-6 protein levels were carried out by commercial ELISA kits (DuoSet ELISA, R&D Systems, Minneapolis, MN) according to the manufacturer’s instructions.

### Measurement of hepatic lipid

To quantify hepatic triglyceride and cholesterol content, liver tissues (100 mg) were homogenized in ice-cold 20 mmol/L Tris–HCl, 150 mmol/L NaCl, 2 mmol/L EDTA, and 1% Triton X-100, pH 7.5. The lipids were extracted by shaking in cold chloroform : methanol (v/v, 2:1) as previously described by Folch et al. [24]. Liver triglyceride and total cholesterol were measured by enzymatic methods using commercial kits (Wako Pure Chemical, Dallas, Texas, USA). The detection limit of triglyceride and total cholesterol were 3 mg/dl.

### Real time quantitative polymerase chain reaction (Real time PCR)

Total RNA of the adipose tissue (for PPARγ, GLUT1, and GLUT4) and hepatic tissue (for Elvol6, FAS, and Srebp-1c mRNA) of each rat were extracted using TRI Reagent (Invitrogen, Carlsbad, CA, USA). Then the extracted RNA was treated with TUBRO DNase (Applied Biosystems, Foster City, CA, USA) for 25 min at 37°C. Two micrograms of TUBRO DNase-treated RNA were reverse transcribed using High Capacity RNA-to-cDNA kit (Applied Biosystems). The cDNA samples were then diluted with nuclease-free water to a final concentration equivalent to 20 ng/μL RNA input, and 6 μL of these dilutions were then subjected to 40 cycles of quantitative Real-time PCR with the SYBR Green Master Mix (Roche Applied Science, Indianapolis, IN, USA) was performed using ABI Prism 7500 Sequence Detection System (Applied Biosystems). Primers for PPAR-γ, sense: 5’-TGCAGGTGATCAAGAAGACG-3’; antisense: 5’-TGGAAGAAGGGAAATGTTGG-3’; GLUT1, sense: 5’-AACTCTTCAGCCAGGGTCCAC-3’; antisense 5’-CACAGTGAAGATGATGAAGAC-3’; GLUT4, sense: 5’-CATGCTGGTCAACAATGTCC-3’; antisense: 5’-GATGTCAGCCCTGAGTAGGC-3’; Elvol6, sense: 5’-ACACGTAGCGACTCCGAAGAT-3’; antisense: 5’-AGCGCAGAAAACAGGAAAGACT-3’; FAS, sense: 5’-GGCATCATTGGGCACTCCTT-3’; antisense: 5’- GCTGCAAGCACAGCCTCTCT-3’, and Srebp-1c, sense: 5' -GGAGCCATGGATTGCACTTT- 3'; antisense: 5' TCAAATAGGCCAGGGAAGTCA 3' were tested alongside the normalizing gene β-actin (sense: 5^′^-TCTGTGTGGATTGGTGGCTCT-3^′^; antisense: 5^′^-GACTC ATCGTACTCCTGCTTGCT-3^′^). The fluorescent signals were collected during extension phase, Ct values of the sample were calculated, and the transcript levels were analyzed by 2^-ΔΔCt^ method.

### Histological analysis

Livers isolated from rats were fixed in 10% formalin and embedded in paraffin. Three-micrometer thick sections were obtained, and deparaffinized in xylene. The sections were stained with hematoxylin-eosin. The results of staining were viewed and photographed with an Olympus microscope (Olympus Corporation, Tokyo, Japan).

### Statistical analysis

Results were expressed as mean ± standard deviation (SD). Statistical analysis was performed using the ANOVA and Duncan’s multiple-range tests. Difference was considered to be significant at p < 0.05.

## Results

### Effects of Lr263 on body weight, food intake, various tissue weights, and homeostasis model assessment-estimated insulin resistance (HOMA-IR) in high-fructose-diet rats

As shown in Table 1, the body weight, liver weight, adipose tissue weight, and HOMA-IR in the HFD group were significantly increased compared to control group. After administration of Lr263, an increase in body weight, liver weight, adipose tissue weight, and HOMA-IR in HFD group were significantly decreased and reached the levels similar to the control group. There were no significant changes in kidney weight and food intake among the three groups.

**Table 1:** **Effects of *Lactobacillus reuteri *GMNL**-**263 on body weight**, **food intake**, **various tissue weights**, **and HOMA**-**IR index in high**-**fructose**-**diet rats**

	**Control**	**HFD**	**HFD**+**Lr263**
Body weight (g)	484.83 ± 29.69	572.67 ± 40.81^#^	502.83 ± 35.91^*^
Food intake (g/day/rat)	28.94 ± 1.35	27.45 ± 1.88	27.54 ± 1.16
Liver weight (% body weight)	2.65 ± 0.26	2.93 ± 0.09^#^	2.53 ± 0.07^*^
Kidney weight (% body weight)	0.71 ± 0.06	0.713 ± 0.01	0.71 ± 0.02
Adipose tissue weight (% body weight)	5.10 ± 0.80	8.29 ± 2.10^#^	6.85 ± 0.16^#^
HOMA-IR	5.24 ± 1.68	19.93 ± 4.51^#^	8.88 ± 1.21^*^

# p<0.05 compared with control group; * p<0.05 compared with HFD group.

### Effect of Lr263 on fecal microflora species in high-fructose-diet rats

*Bifidobacteria* and *Lactobacilli* have been regarded as beneficial microflora species, whereas some species, *Clostridia* for example, would be harmful as a consequence of their metabolic activities. The number of *Bifidobacteria*, *Lactobacilli*, and *Clostridia* in control group were regarded as 100% for comparison. Our present results indicated that the number of *Bifidobacteria*, *Lactobacilli*, and *Clostridia* in HFD group were 103.2 ± 6.4%, 97.4 ± 3.8%, and 130.1 ± 5.9%, respectively. These results revealed that no significant change on the number of *Bifidobacteria* and *Lactobacilli* in HFD group was noticed as compared to control group. However, the number of *Clostridia* in HFD group was significantly higher than that of control group. After 14 weeks of Lr263 treatment, the number of *Bifidobacteria*, *Lactobacilli*, and *Clostridia* were 126.7 ± 3.4%, 135.4 ± 7.8%, and 102.5 ± 4.9%, respectively. These results demonstrated that administration of Lr263 significantly increased the number of *Bifidobacteria* as well as *Lactobacilli* and decreased the number of *Clostridia* when compared to HFD group.

### Effects of Lr263 on oral glucose tolerance test (OGTT) in high-fructose-diet rats

**Figure 1:**
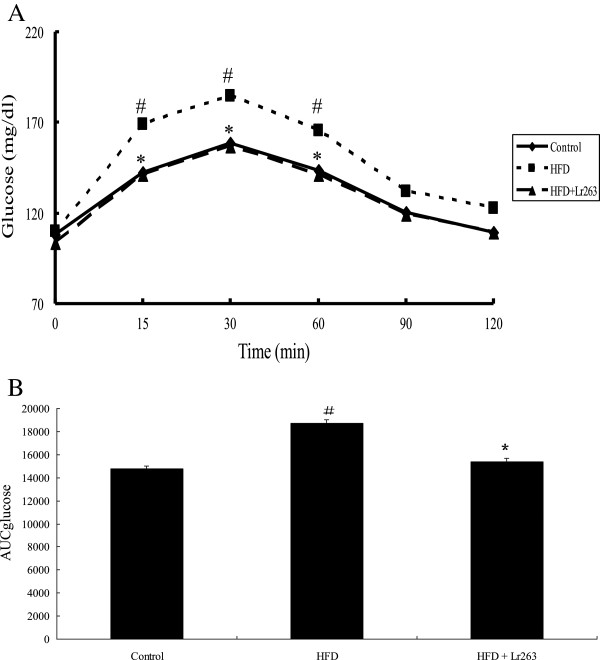
**Effect of *Lactobacillus reuteri *GMNL-263 on oral glucose tolerance test (OGTT) in high-fructose-diet rats for 14 weeks.** The test was performed at 0, 4, 8, and 14 weeks. The results of OGTT at 14 weeks after treatment were shown in (**A**). AUC_glucose_ value which represented the area under the curve of glucose level at 14 weeks after treatment was shown in (**B**). Data were expressed as mean ± SD (n = 6). Control, control group; HFD, high fructose diet group; HFD + Lr263, high fructose-treated and *Lactobacillus reuteri* GMNL-263 group. #p < 0.05 compared with Control group; *p < 0.05 compared with HFD group.

The results of OGTT after 14 weeks treatment were shown in Figure 1. There were no significant differences on blood glucose levels among the three groups within 8 weeks after treatment (data not shown). However, a marked increase in blood glucose concentrations at 15, 30, and 60 min after glucose loading in HFD group were noticed. This increase in serum glucose levels in HFD group was significantly decreased after administration of Lr263 (Figure 1A). The AUC_glucose_ values shown in Figure 1B clearly reflected the inhibitory effect of Lr263 on blood glucose elevation by high fructose treatment.

### Effects of Lr263 on serum levels of glycated haemoglobin, glucose, insulin, leptin, C-peptide, and plasma levels of active GLP-1 and total GIP in high-fructose-diet rats

**Figure 2:**
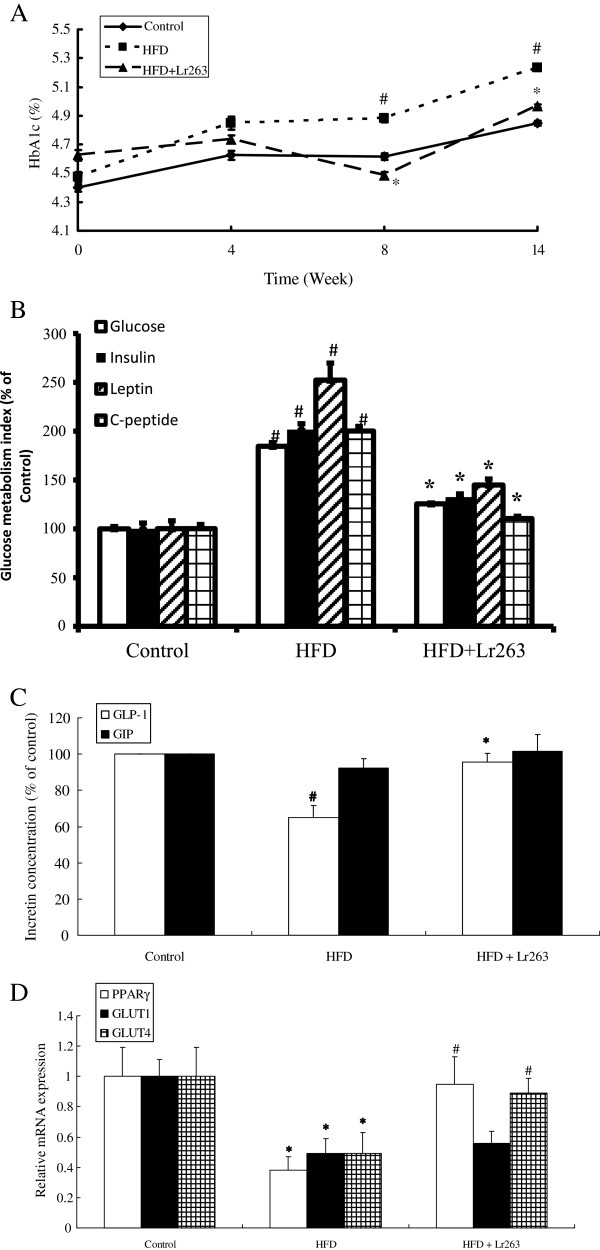
***Lactobacillus reuteri *GMNL-263 ameliorates insulin resistance and facilitates glucose uptake in high-fructose-diet rats.** Effects of *Lactobacillus reuteri* GMNL-263 on glycated hemoglobin (HbA1c) (**A**), glucose metabolism index (including serum glucose, insulin, leptin, and C-peptide levels) (**B**), plasma incretin (active GLP-1 and total GIP) concentrations (**C**), and insulin sensitivity regulation as well as glucose uptake related genes expression in adipose tissue (PPARγ, GLUT1 and GLUT4) (**D**) in high-fructose-diet rats. The serum glucose metabolism index, plasma incretin concentrations, and relevant mRNA were measured at 14 weeks after treatment. The whole blood samples were used for determination of glycated hemoglobin (HbA1c) at week 0, 4, 8, and 14. Values were expressed as mean ± SD (n = 6). #p < 0.05 compared with Control group; *p < 0.05 compared with HFD group.

The levels of HbA1c, an important indicator for glucose metabolism, were markedly increased at 8 and 14 weeks in HFD group when compared to control group. However, the elevations of HbA1c in HFD group at 8 and 14 weeks were downregulated by administration of Lr263 (Figure 2A). In addition, the fasting serum concentrations of glucose, insulin, leptin, and C-peptide were significantly higher in HFD group than those of control group. However, the increase in serum concentrations of glucose, insulin, leptin, and C-peptide in HFD rats were significantly decreased after administration of Lr263 (Figure 2B). As shown in Figure 2C, a significant decrease in active GLP-1 was observed in HFD groups as compared to control group. Lr263 administration significantly stimulated active GLP-1 expression. However, the plasma concentration of total GIP, another important member of incretin, was not significantly different among the control, HFD, and HFD + Lr263 groups.

### Effect of Lr263 on hepatic injury markers and antioxidant activities in high-fructose-diet rats

**Figure 3:**
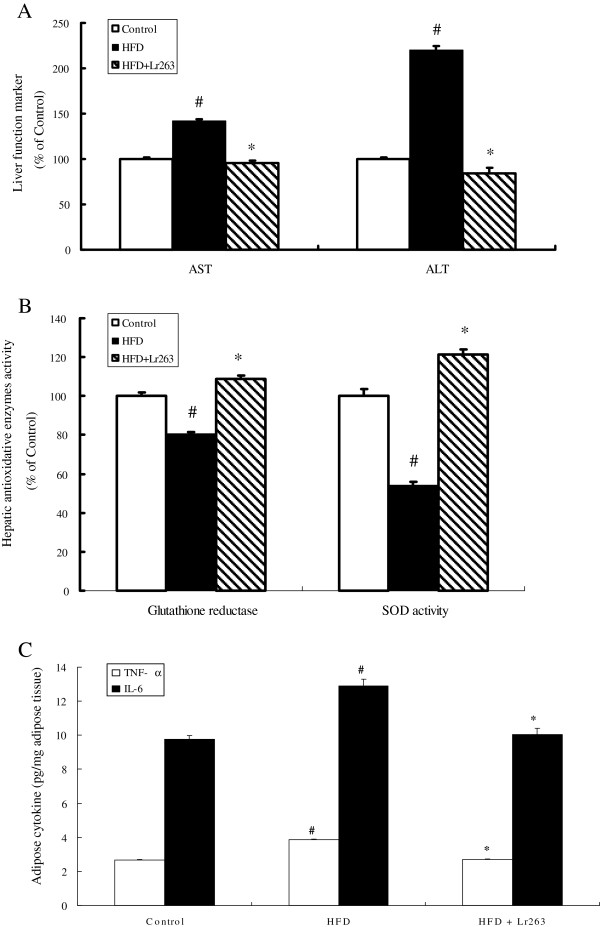
***Lactobacillus reuteri *GMNL-263 improves liver injury and oxidative stress in high-fructose-diet rats.** Effects of *Lactobacillus reuteri* GMNL-263 on expressions of liver injury markers (AST and ALT) (**A**), hepatic enzymatic activities of antioxidant (SOD and GR) (**B**), and pro-inflammatory cytokines (TNF-α and IL-6) (**C**) in high-fructose-diet rats at the end of the experimental period (14 weeks). The data were expressed as mean ± SD (n = 6). SOD, speroxide dismutase; GR, glutathione reductase. AST, aspartate aminotransferase; ALT, alanine aminotransferase; TNF-α, tumor necrosis factorα; IL-6, interleukin 6. #p < 0.05 compared with Control group; *p < 0.05 compared with HFD group.

Increased serum levels of AST and ALT were found in HFD group (Figure 3A). These results showed that long-term high fructose treatment induced damage to liver cells. However, administration of Lr263 significantly lowered the increased AST and ALT levels in HFD group. The results shown in Figure 3B revealed that the activities of SOD and glutathione reductase in rat livers were decreased, respectively, as compared to control group. Administration of Lr263 markedly upregulated these two antioxidant activities, which were decreased in HFD group, in rat livers (Figure 3B).

### Effect of Lr263 on adipose tissue inflammatory cytokines in high-fructose-diet rats

Two important insulin resistance-related inflammatory cytokines namely TNF-α and IL-6 produced by adipose tissue were evaluated by commercially available ELISA kit. As shown in Figure 3C, the concentrations of TNF-α and IL-6 in HFD group were significantly enhanced as compared to control group, and this enhancing effect was markedly abrogated by Lr263 treatment (HFD + Lr263 group).

### Effect of Lr263 on serum and hepatic lipid profile in high-fructose-diet rats

**Figure 4:**
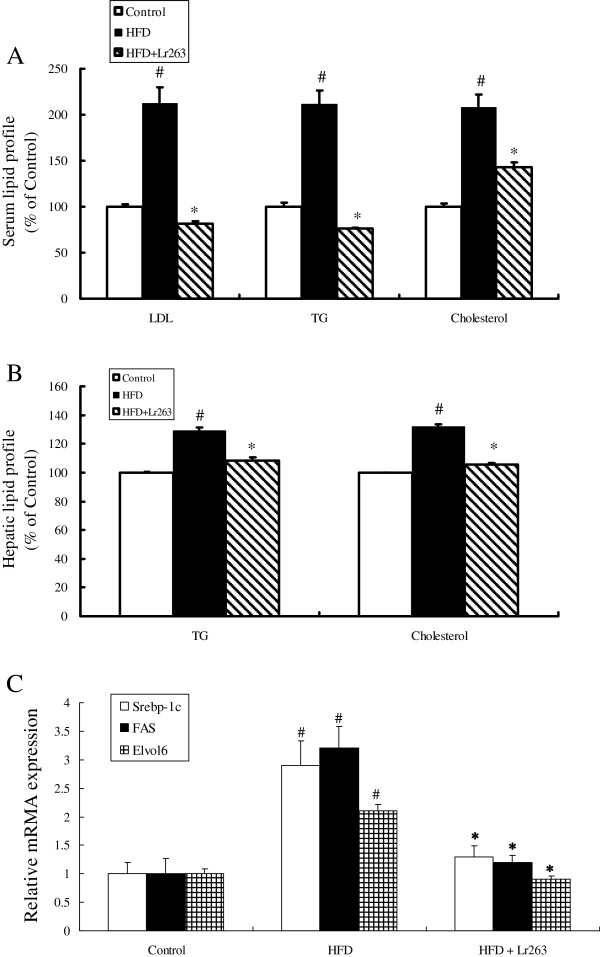
***Lactobacillus reuteri *GMNL-263 attenuates lipogenesis in high-fructose-diet rats.** Effects of *Lactobacillus reuteri* GMNL-263 on expressions of serum lipid profiles (LDL, TG and cholesterol) (**A**), hepatic lipid profile (TG and cholesterol) (**B**), and hepatic lipogenic genes (Elvol6, FAS, and Srebp-1c) (**C**) in high-fructose-diet rats at 14 weeks after treatment. The results were expressed as mean ± SD (n = 6). LDL, low-density lipoprotein; TG, triglyceride; Elvol6, fatty acid elongase 6; FAS, fatty acid synthase; Srebp-1c, sterol regulatory element-binding protein 1c. #p < 0.05 compared with Control group; *p < 0.05 compared with HFD group.

Previous studies indicated that increased levels of TG and cholesterol were the main predictors and causative factors for inducing insulin resistance in type 2 diabetes [25]. In our current results, higher serum levels of LDL, TG, and cholesterol were noticed in HFD group as compared to control group (Figure 4A). Administration of Lr263 significantly reduced the increased levels of LDL, TG, and cholesterol seen in HFD group (Figure 4A). To examine whether dietary Lr263 can suppress the formation of hepatic steatosis, the levels of TG and cholesterol in rat liver cells were performed. Our results demonstrated that higher levels of hepatic TG and cholesterol were found in HFD group as compared to control group. Feeding of Lr263 significantly suppressed the increased hepatic TG and cholesterol levels in HFD group (Figure 4B).

### Effect of Lr263 on PPARγ, GLUT1, GLUT4, and hepatic lipogenic gene expressions in high-fructose-diet rats

The PPARγ mRNA, GLUT1 mRNA, and GLUT4 mRNA expression levels in adipose tissue were shown in Figure 2D. The PPARγ mRNA, GLUT1 mRNA, and GLUT4 mRNA levels in adipose tissue were significantly decreased in HFD group compared with control group. Additionally, following the treatment with Lr263 (HFD + Lr263 group), PPARγ mRNA and GLUT4 mRNA expressions were significantly increased compared with HFD group. However, the expression of GLUT1 mRNA showed no significant change between HFD group and HFD + Lr263 group. Expression of genes involved in hepatic fatty acid synthesis (Elvol6, FAS, and Srebp-1c) were induced in rats fed with high fructose diet (HFD group) compared to control group. Administration of Lr263 (HFD + Lr263 group) significantly reduced the increased mRNA expressions to near control levels (Figure 4C).

### Effect of Lr263 on hepatic steatosis by histological analysis

**Figure 5:**
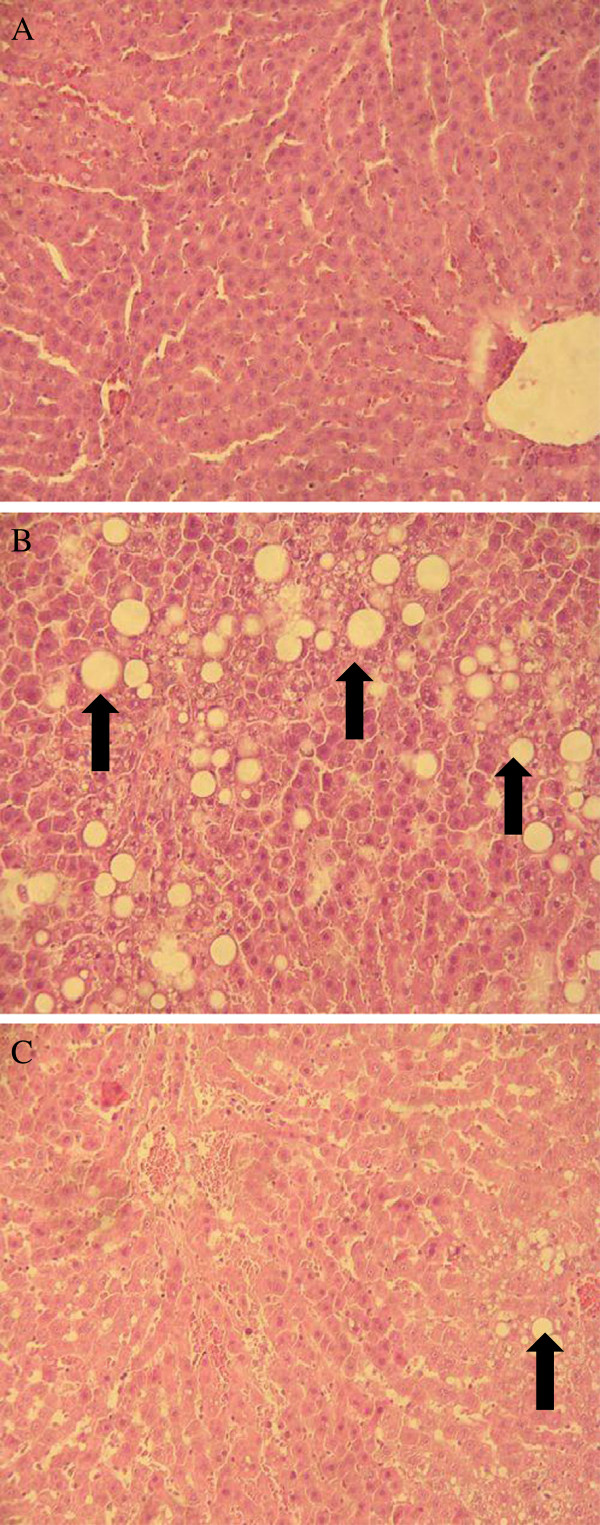
**Effects of *Lactobacillus reuteri *GMNL-263 on hepatic steatosis in treated rats.** At the end of the experimental period (14 weeks), the livers obtained from sacrificed rats were subjected to hematoxylin and eosin staining for evaluation of hepatic steatosis. The histological sections of rat liver were observed at magnification 200 ×. (**A**) Liver section from control rat showed normal appearance of liver cells; (**B**) Liver section from high-fructose-diets fed rats showed marked fatty infiltration of hepatocytes with macro- or micro-vesicular steatosis (black arrow); (**C**) Liver section from *Lactobacillus reuteri* GMNL-263 and high fructose-treated rats showed mild fatty infiltration of hepatocytes (black arrow). The result shown here was from one representative experiment of six different samples with similar results.

In order to verify the efficacy of Lr263 on prevention of development of hepatic steatosis in rats treated with high fructose, the histological analysis of rat liver tissues were performed. As demonstrated in Figure 5, the results of staining showed prominent diffuse macro-vesicular steatosis in liver obtained from the HFD group (Figure 5B) compared to the normal histological appearance of the liver from control group (Figure 5A). However, administration of Lr263 significantly reversed the formation of hepatic steatosis induced by high fructose treatment (Figure 5C).

## Discussion

Recent studies on new medications approved for the treatment of type 2 diabetes are focused on various strategies to prevent and/or delay the onset of type 2 diabetes and its complications [19,26]. One of these strategies is the consumption of foods which are low in the glycemic index with bioactive agents that have been adopted to prevent or delay the onset of disease [26]. Administration of probiotics has been reported to be one of the most widely used approaches to modulate intestinal microbiota and may subsequently prevent or delay diabetes incidence [27]. Cani et al. [28] demonstrated that *Bifidobacterium* species improved glucose tolerance, glucose-induced insulin secretion, normalized inflammatory tone, and reduced the incidence of diabetes. Strains of lactic acid bacteria are the most common microbes employed as probiotics. In addition, most probiotic strains belong to the genus *Lactobacillus*. Several lines of evidence indicated that some beneficial effects on human health were associated with the intake of lactic acid bacteria [29]. Recently, it has been reported that lactic acid bacteria have efficacy relating to the progression of diabetes [20,30]. Previous reports demonstrated that *Clostridium* species could trigger the inflammatory process in human inflammatory bowel diseases [31]. Furthermore, studies from Montesi et al. [22] demonstrated that the diet containing probiotics was able to modify the bacterial composition of the caecal content in rats and to induce a reduction in *clostridium* species, a bacterial group that was generally considered to be “non-desirable” members of the intestinal microbiota. Moreover, a significant reduction in *Clostridium* species in human fecal analysis was strongly associated with improving on some metabolic diseases [32]. As shown in our studies, Lr263 consumption significantly increased the number of *bifidobacteria* and *lactobacilli*, and on the contrary, decreased the number of *Clostridia* in the feces of treated rats when compared to those of the HFD rats. These results implicated that Lr263 administration might exert its therapeutic effect on diabetes via increasing the beneficial as well as decreasing the harmful gut flora species. However, the relevant mechanisms underlying this phenomenon needed further investigation.

Previous study revealed that increased weight gain and fat deposition were responsible for insulin resistance [33]. In addition, a number of studies reported that a reduction of visceral fat in animal models and humans was associated with increased insulin sensitivity [34]. Our results shown in Table 1 demonstrated that the body weight, liver weight, and adipose tissue weight in HFD group were significantly higher than those of control group. These results were in line with previous reports [25]. Administration of Lr263 significantly suppressed the enhancing effect of high-fructose treatment on body weight, liver weight, and adipose tissue weight. These results implied that reduction of body weight, liver weight, and adipose tissue weight by Lr263 treatment may be responsible for improving insulin sensitivity in HFD rats. Insulin resistance, determined by HOMA-IR index, was significantly higher in HFD rats when compared to control group. However, Lr263 consumption significantly abrogated the increased HOMA-IR index in HFD rats, further corroborating the effectiveness of Lr263 on improvement of insulin resistance (Table 1).

Impaired glucose tolerance testing is an important diagnostic indicator for type 2 diabetes [35]. Our results from OGTT indicated that an increase in blood glucose level was observed after high-fructose-diet feeding. Administration of Lr263 significantly decreased the blood glucose levels enhanced by high-fructose treatment. These results demonstrated that feeding of Lr263 may improve glucose intolerance and prevent the development of hyperglycemia in high-fructose-induced diabetic rats. HbA1c is an important factor used to monitor the long-term blood glucose balance, because it reflects the number of glucose molecules attached to hemoglobin in red blood cells. Similarly, while the HbA1c levels were increased in HFD rats, feeding Lr263 significantly downregulated the HbA1c levels enhanced by high fructose treatment. These results further corroborated the findings in OGTT.

In the obese state, an increase in adipose tissue mass leads to increased secretion of insulin and leptin into the circulation [36] resulting in the development of hyperinsulinemia and hyperleptinemia, respectively [37,38]. Insulin and C-peptide are co-secreted from the β-cell in a 1:1 ratio [39]. Unlike insulin, C-peptide has negligible extraction by the liver and constant peripheral clearance. Therefore, C-peptide is commonly used in preference to insulin measurement when assessing β-cell function in clinical practice [40]. As shown in our current results, the weights of adipose tissue in HFD rats were higher than those of control rats which may result in the increased production of insulin, C-peptide and leptin in HFD rats seen in our study. Lr263 feeding abrogated the elevations of glucose, insulin, C-peptide and leptin levels in HFD rats. Moreover, previous study indicated that increased leptin was positively associated with hyperinsulinemia and insulin resistance [41]. Therefore, it was reasonable to propose that leptin resistance may act synergistically with insulin resistance to result in type 2 diabetes development. Our results suggested that administration of Lr263 could prevent the development of type 2 diabetes by normalizing serum levels of glucose, insulin, C-peptide and leptin. It has long been recognized that some bioactive agents (hormones) produced by the gastrointestinal system can modulate the secretory activities of the islets of Langerhans. These bioactive agents stimulating the greater insulin secretion from pancreatic beta cells in response to oral glucose were referred to as incretins [42]. Two important incretins namely GIP and GLP-1 secreted by K cells and L cells in the small intestine, respectively, have been shown to account for as much as 70% of the insulin secretion immediately after meal ingestion [43]. Our present results showed that the secretion of GLP-1 in HFD group was significantly lower than that of control group. Administration of Lr263 upregulated the decreased GLP-1 level caused by high fructose treatment. However, the production of GIP among the control, HFD, and HFD + Lr263 groups were not significantly different. Previous studies indicated that augmentation of GLP-1 action was widely used for the treatment of type 2 diabetes. Furthermore, GLP-1 not only acted as an incretin to lower blood glucose via stimulation of insulin secretion from islet β cells but also inhibited gastric emptying and acid secretion, reduced food ingestion and glucagon secretion [44]. Vilsboll and coworkers [45] showed that a normal GIP secretion, but reduced postprandial concentrations of biologically active GLP-1 in type 2 diabetic patients. Previous studies have indicated that whereas GLP-1 is strongly insulinotropic in patients with Type II diabetes mellitus, the effect of GIP is much weaker or absent [46,47]. In fact, GLP-1 was reported to play a more important role on normalizing fasting hyperglycemia [48]. Therefore, our present findings suggested that the effect of Lr263 on improving hyperglycemia in HFD rats was mostly attributed to GLP-1 secretion.

Oxidative stress is considered to play an important role in the pathogenesis of type 2 diabetes [49]. In addition, oxidative stress is also associated with progression of hepatic steatosis and advanced liver damage resulted from degenerative changes in the enzymatic antioxidant defense system [50,51]. In our present data, the serum levels of AST and ALT, two critical markers of liver injury, were increased in HFD rats; meanwhile, the activities of hepatic antioxidants namely SOD and GR were decreased in HFD rats. These results demonstrated that high fructose treatment exhibited deleterious effects on antioxidant production and subsequently enhanced oxidative stress in rats, which then resulted in liver damage in HFD rats seen in our study. After treatment with Lr263, downregulation of serum AST and ALT levels and upregulation of hepatic activities of SOD and GR in HFD rats were noticed. These findings implied that Lr263 consumption significantly reduced both oxidative stress and liver damage in HFD rats via enhancing hepatic antioxidants expressions. There is considerable evidence that hyperglycemia results in the generation of reactive oxygen species (ROS), ultimately leading to increased oxidative stress in a variety of tissues. Oxidative stress is supposed to stimulate the production of a variety of proinflammatory cytokines, which may ultimately lead to insulin resistance [52-54]. Both TNF-α and IL-6, two main proinflammatory cytokines released by adipose tissue, can inhibit insulin signalling, and TNF-α may have a crucial role in the development of insulin resistance in type 2 diabetes [55]. Our present study revealed that Lr263 administration markedly suppressed the increased TNF-α and IL-6 production by adipose tissue in HFD rats, suggesting that Lr263 ameliorated insulin resistance through reducing oxidative stress as well as proinflammatory cytokines production.

The major pathogenesis of metabolic syndrome is the development of insulin resistance caused by the accumulation of visceral fat which promoted the elevation of blood pressure, dyslipidemia, and dysregulation of glucose metabolism [56]. Among the target tissues of insulin, liver is the principal regulator of glucose and lipid metabolism by controlling hepatic glucose production, glycogen storage, and lipogenesis [56]. As shown in our study, administration of Lr263 dramatically decreased the levels important components of metabolic syndrome, including serum glucose, insulin, LDL, TG, and cholesterol, enhanced by high fructose treatment. In addition to serum levels, the increased hepatic levels of TG and cholesterol treated with high fructose were also found to be suppressed by oral administration of Lr263. In present study, our data implicated that administration of Lr263 could improve insulin resistance via downregulation of both serum and hepatic lipid concentrations. To elucidate the possible mechanisms of Lr263 on suppression of hepatic lipid accumulation in HFD rats, we assessed liver mRNA levels of genes involved in lipogenesis. Excess fructose consumption is closely associated with the development of type 2 diabetes due to insulin insensitivity and dyslipidemia. Srebp-1c is a well-known transcription factor that regulates hepatic fatty acid and triglyceride biosynthesis by upregulating the expression of key genes, such as FAS [57]. In addition, dietary fructose markedly increases the expression of hepatic Srebp-1c, and FAS in rats [58]. Elvol6 is an elongase that specifically catalyzes the elongation of saturated and monounsaturated fatty acids with 12, 14, and 16 carbons [59]. Numerous studies have demonstrated that Elvol6 regulated by SREBP-1, playing an important role in de novo synthesis of long-chain saturated and monounsaturated fatty acids in conjunction with FAS [60]. The findings of our study showed that high fructose treatment markedly induced lipogenic genes namely s rebp-1c, FAS, and Elvol6 expressions in rat liver. Administration Lr263 counteracted the increase in hepatic lipogenic genes in HFD rats. These results suggested that Lr263 consumption not only suppressed hepatic lipid accumulation, but also subsequently improved insulin resistance in HFD rats.

In contrast to type 1 diabetes, in which there is an absolute insulin deficiency due to destruction of islet cells in the pancreas, type 2 diabetes is a metabolic disorder that is characterized by high blood glucose in the context of insulin resistance and relative insulin deficiency. PPAR-γ, a nuclear receptor, plays a critical role in glucose and lipid metabolism. Moreover, PPAR-γ also plays an important role in regulating insulin sensitivity and glucose homeostasis [61]. Glucose transporter proteins, GLUT1 and GLUT4, the two integral isoforms present in adipose tissue, play the role of tissue glucose uptake and regulate body glucose homeostasis [62]. While GLUT1 is involved in a low intensity of glucose uptake, GLUT4 plays a critical role in regulation of glucose homeostasis and functions as a key modulator of glucose disposal in fat [63]. PPAR-*γ* activity has been shown to directly regulate the expression of GLUT4 involving in regulating insulin-stimulated glucose transport [64]. Our study demonstrated that a decrease in PPAR-γ mRNA expression in adipose tissue of HFD rats was observed. However, Lr263 administration restored the PPAR-γ mRNA expression reduced by high fructose treatment. In addition, both GLUT1 and GLUT4 mRNA expressions in adipose tissue of HFD rats were lower than those of control group. While GLUT1 mRNA expression remained unchanged, the level of GLUT4 mRNA was significantly enhanced by Lr263 treatment. These results implicated that Lr263 may improve insulin resistance, enhance glycemic control, and maintain glucose homeostasis in adipose tissue through upregulation of PPAR-γ and GLUT4 expressions.

Insulin resistance plays a crucial role in hepatic steatosis [65], which is closely associated with obesity and often accompanied by marked abdominal adiposity [66]. Fat accumulation in the liver and insulin resistance cause as well as potentiate each other, and creating a vicious cycle of metabolic dysfunction resulting in the development of hepatic steatosis [67]. Our results from histological analysis demonstrated that a prominent hepatic steatosis was observed in HFD rats, whereas administration of Lr263 significantly ameliorated fat accumulation in liver as compared to HFD group. These findings indicated that hyperlipidemia and insulin resistance caused by high fructose treatment were improved by administration of Lr263, which may ameliorate the development of hepatic steatosis in HFD rats.

## Conclusion

Previous studies regarding the therapeutic effectiveness of *Lactobacillus* species on diabetes and its complications were focus on increasing insulin sensitivity [68] or delaying the onset of glucose intolerance, hyperglycemia, hyperinsulinemia, dyslipidemia, and oxidative stress [17,19,69]. In line with previous studies, oral administration of Lr263 significantly improved insulin resistance, glucose intolerance, oxidative stress, fatty liver, and hepatic damages in rats fed with high fructose diet. The results presented herein not only provided a solid theoretical basis regarding how Lr263 exhibited its effectiveness on treating diabetes, but also shed light on the development of Lr263 as a promising therapeutic option for the management of type 2 diabetes.

## Competing interests

All authors declare no competing interests.

## Authors’ contributions

C-SW was responsible for the study design and manuscript preparation; F-CH and C-LL performed animal experiments; C-YC and W-TC contributed to the immunohistochemical staining of liver tissues; C-SW and F-CH contributed to the manuscript writing, data processing, interpretation, and analyses. All authors have read and approved the final manuscript.
